# Change in Prevalence of Hepatitis B Virus Infection in Pregnant Women in the Last Two Decades in Thailand

**DOI:** 10.3390/v16020314

**Published:** 2024-02-19

**Authors:** Yosagorn Porngasemsart, Sirinart Sirilert, Theera Tongsong

**Affiliations:** Department of Obstetrics and Gynecology, Faculty of Medicine, Chiang Mai University, Chiang Mai 50200, Thailand; yosagorn_p@cmu.ac.th (Y.P.); theera.t@cmu.ac.th (T.T.)

**Keywords:** hepatitis B virus, pregnant women, prevalence, Thailand

## Abstract

***Objectives:*** In Thailand, there has been a strategy to prevent the mother-to-child transmission of HBV for over 30 years. However, there is still a lack of empirical evidence regarding the effectiveness of this strategy. This study aims to investigate the trends in the prevalence of HBV infection in pregnant women and to identify factors that may be associated with the prevalence of HBV infection in pregnant women. ***Patients and Methods:*** A maternal–fetal medicine database was accessed to retrieve the consecutive obstetric records of women giving birth at Chiang Mai University Hospital, Thailand, from January 2003 to December 2022. All women undergoing HBV tests with available results were included for an analysis of the trends and changes in the prevalence of maternal HBV infection. Also, the rates of infection in different age cohorts were compared. ***Results:*** During the study period, a total of 36,958 women were eligible for analysis. Overall, the prevalence of HBV infection in pregnant women was found to be 5.3% (1970 cases). Overall, HBV prevalence fell from 6.11% in 2003 to 3.15% in 2022. There was a significant reduction, especially in the adolescent group, decreasing from 8.26% in 2003 to 0% in 2022. In the reproductive age group, the prevalence significantly decreased from 6.41% to 2.01%. However, the prevalence in the elderly group was unchanged. The only significant risk factor was the years in the early timeline of the study period, presumably associated with previous HBV vaccination. Other factors, such as socioeconomic status, residential area, and being a private case, were not correlated with the prevalence of HBV. ***Conclusion:*** The prevalence of HBV infection in pregnant women has significantly decreased in the past two decades from 6.11% in 2003 to 3.15% in 2022. The percentage of reduction was very striking in the adolescent group, dropping from 8.6% in 2003 to 0.0% in 2022 or being nearly eradicated in the most recent years. Our results suggest that the overall prevalence of HBV infection among our pregnant women will probably be less than 1.0% in the near future.

## 1. Introduction

Infection with the hepatitis B virus (HBV) affects people all over the world, involving approximately 257 million people or accounting for 3.5% of the world population. Maternal-to-child transmission or vertical transmission of HBV is of significant concern, often resulting in chronic HBV infection, which can progress to cirrhosis or hepatocellular carcinoma. Also, hepatitis B virus infection is recognized as the seventh leading cause of morbidity and mortality globally [1,2]. The World Health Organization (WHO) has set a target to eliminate HBV infection by 2030, striving for a prevalence of less than 0.1% in children under 5 years old [3]. Nevertheless, although maternal screening and neonatal immunoprophylaxis can reduce the prevalence of vertical transmission from 25–90% [4,5,6,7] to 1–4% [8,9,10,11,12,13], 50 million new cases of HBV infection are still diagnosed every year. The majority of these new cases are caused by breakthrough infection and the failure of neonatal immunoprophylaxis, resulting in vertical transmission [14]. Enormous evidence indicates that a high level of HBV viral DNA is a high-risk factor for vertical transmission and immunoprophylaxis failure in children born to HBV-infected mothers [15]. To achieve the goal set by the WHO, various strategies have been employed, including universal neonatal HBV vaccination, screening for HBV infection in all pregnant women, administration of antiretroviral drugs to reduce HBV viral load in high-risk individuals, and provision of HBV immunoglobulin (HBIG) to infants born to HBV-infected mothers [13,16,17]. In the evaluation of the effectiveness of such preventive measures, the data concerning changes in the prevalence of HBV in various populations in recent years are important for surveillance, strengthening, and reinforcement. In addition to the overall prevalence in the population, changes in the prevalence in target groups of special concern, like pregnant women, are extremely important, especially in areas where mother-to-child transmission is the main route of transmission. These data of actual trends in the real world can help us modify or strengthen the strategy of prevention and control properly. The prevalence changes among pregnant women are more useful than the overall prevalence in the estimation of the number of new cases of infected newborns in the near future because mother-to-child transmission is the most important transmission in Thailand. The prevention strategy in this group is different from the general population and needs special measures involving both the effectiveness of screening for women with HBV-positivity and the availability of passive HBV immunoglobulin for newborns, as well as viral load determination and antiviral drug administration in cases of high viral levels, as such factors impact on prophylaxis failure. Accordingly, the prevalence and trends of HBV infection among pregnant women are crucial as vital determinants of the effectiveness of policy in the prevention and prediction of the number of new cases in the near future, as well as for use in modifying the strategy of prevention suitable for each population.

In Thailand, the HBV vaccine has been incorporated into the Expanded Program on Immunization (EPI) for all neonates and infants since 1992 [18,19]. The strategy appears to have been successful in lowering the prevalence of HBV infection in children. However, epidemiologic studies show that prevalence was still high in adults, with 3.48% in a large series in Thailand in 2016 [20] and 5.1% in a systematic review in Thailand in 2016 [21]. At Maharaj Nakorn Chiang Mai Hospital, a policy following the national guideline has been in effect since 2000 to prevent maternal-to-child HBV transmission by screening all pregnant women. This involves routine testing for the HBV surface antigen (HBsAg) in asymptomatic cases. Additionally, vaccination and HBV immunoglobulin (HBIG) are administered to infants born to HBV-infected mothers within 12 h of birth. Since 2015, viral load testing has been implemented, and antiretroviral therapy has been initiated for mothers with high virus levels (more than 200,000 IU/mL) from 24 to 32 weeks of gestation until delivery or 1–3 months after delivery. Despite the implementation of this policy for more than two decades aimed at reducing HBV transmission from mothers to infants, there has been no empirical assessment of its outcomes. Therefore, we conducted this study, which aimed to identify the trends in the prevalence of HBV infection over the past two decades in all pregnant women and subgroups based on maternal age. This investigation is critical for evaluating the effectiveness of the longstanding policy in the context of high HBV prevalence in countries like Thailand and its potential impact on reducing chronic liver disease and liver cancer.

## 2. Patients and Methods

This trend analysis study was carried out based on the obstetric database of the Department of Obstetrics and Gynecology, Chiang Mai University, Thailand. The computerized database of pregnant women who had their babies delivered at our hospital, a tertiary medical training center taking care of people in both urban and rural areas in northern Thailand, was developed in 1990. Each patient included in the database was prospectively entered consecutively on the day of their discharge from the hospital. Each record included demographic as well as clinical data (e.g., maternal age, parity, residency, socioeconomic status, underlying medical diseases, etc.) and obstetric data (e.g., obstetric complications, such as fetal growth restriction, gestational age at delivery, route of delivery, neonatal outcomes, etc.). This study was ethically approved by the Institutional Review Board, Faculty of Medicine, Chiang Mai University, Research ID: study code OBG-2566-0375 and complied with the Declaration of Helsinki.

The investigation consolidated data derived from the entire cohort of pregnant women who had their babies delivered at our hospital from January 2003 to December 2022. During the study period, all pregnant women were routinely screened for HBV infection at their first antenatal care visit. The newborns of HBV-negative mothers received the HBV vaccine within 12 h after birth. Those of HBV-positive mothers received the HBV vaccine and HBIG within 12 h after birth. During the last 7 years (2015 to 2022), we also tested for HBeAg (hepatitis B virus e-antigen), measured the viral load levels in the HBeAg-positive pregnant women, and provided antiretroviral therapy (tenofovir) for mothers with high HBV DNA levels (more than 1,000,000 copies/mL) from 24 to 32 weeks of gestation until 4 weeks after delivery or 1–3 months after delivery.

The database was accessed to retrieve the consecutive records of the women meeting the inclusion criteria, including all women screened for HBsAg with known results. The data incorporated in this study encompassed baseline characteristics, HBV status, and potential risk factors for HBV infection, including variables such as residential area, age, and parity. The records were validated by the authors, and full medical records were comprehensively reviewed in some cases as needed. Subsequently, the study cohort was dichotomized into two subsets: individuals with and without HBV infection. Within each subset, subjects were further stratified based on maternal age into three categories: (1) adolescent (age < 20 years), (2) an adult group (age 20–34 years), and (3) an elderly group (age ≥ 35 years).

The main outcome was the trends of prevalence of HBV infection in the past two decades in all women and specific age groups. The secondary outcome was an assessment of the risk factors associated with the prevalence of HBV infection in pregnancy.

### Statistical Analysis

The data analysis was carried out utilizing the Statistical Package for the Social Sciences (SPSS), version 26.0 (IBM Corp., Released 2019, Armonk, NY, USA). Descriptive statistics, represented as mean ± standard deviation, were employed to characterize demographic data. Student’s *t*-test or Mann–Whitney U test was applied for the comparison of means or median in various continuous variables, as appropriate. To assess trends over time for continuous variables, one-way ANOVA served as a time series trend test. Poisson regression was used to assess trends over time for prevalence. Chi-square statistics were used for the comparison of frequency distributions in categorical variables across subgroups. Univariate and multivariate (logistic regression) analyses were undertaken to explore the potential risk factors associated with HBV, including private cases, study period, parity, age groups, residency, and socioeconomic levels. Statistical significance was established at a *p*-value less than 0.05.

## 3. Results

**Figure 1:**
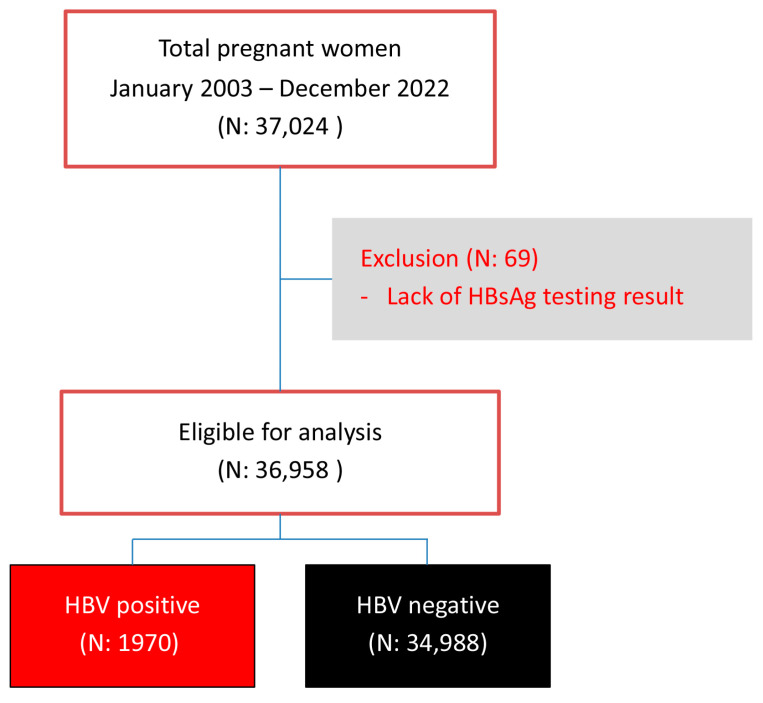
Flowchart of patient enrollment.

**Figure 2:**
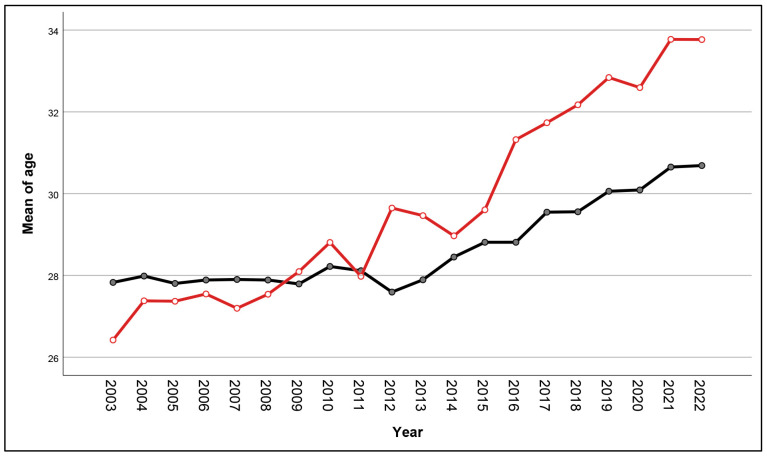
Trend analysis of mean maternal age of women with (red line) and without (black line) HBV infection according to the years of delivery.

During the study period, a total of 37,027 pregnant women attended antenatal care and gave birth at our center. Of them, 99.8% were tested for HBV infection, and 69 women were excluded because of no test results. Therefore, the remaining 36,958 women were available for analysis, including 1970 HBV-positive women and 34,988 HBV-negative women, as presented in Figure 1. It was observed that the mean maternal age significantly increased in both groups. Maternal age in each year was distributed normally (Kolmogorov–Smirnov test: *p*-value > 0.05). In the non-HBV-infected group, the mean age increased from 27.8 ± 5.9 in the year 2003 to 30.7 ± 5.4 in the year 2022 (*p*-value < 0.001), whereas, in the HBV-infected group, it increased from 26.4 ± 5.8 in the year 2003 to 33.8 ± 4.9 in the year 2022 (*p*-value < 0.001). Notably, the increase in maternal age in the HBV-infected group was significantly higher than that in the noninfected group (one-way ANOVA trend analysis; *p*-value < 0.001), suggesting that women with HBV infection were predominantly found in the older age group, as presented in Figure 2.

**Figure 3:**
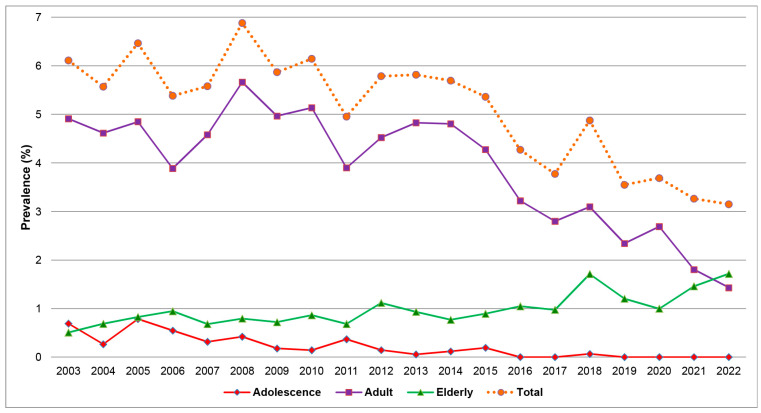
Prevalence of HBV infection in total and subgroups of maternal age by the years of delivery.

Table 1 and Figure 3 show the prevalence of HBV-positive patients in each year, revealing a statistically significant trend of decline from 6.11% in 2003 to 3.15% in 2022 (Poisson regression; Wald–chi-square: 72.973, *p*-value < 0.001). Further subgroup analysis by age group indicates a substantial decrease in the prevalence among the adolescent group, with a statistically significant reduction trend from 8.26% to 0% (Poisson regression; Wald–chi-square: 36.844, *p*-value 0.005). (The number of positive cases has been zero since 2019.) In the reproductive age group, there was also a significant decline in the prevalence from 6.41% in 2003 to 2.01% in 2022 (*p*-value < 0.001). Conversely, the prevalence in the elderly group tended to increase from a prevalence of 3.38% in 2003 to 6.47% in 2022. However, such a change was not statistically significant (*p*-value = 0.623). Note in Figure 3 that the overall prevalence in the adolescent and elderly groups, when based on the total obstetric population, was lower than the adult group because these two groups account for a small portion of the total population.

Based on univariate analysis, as presented in Table 2, it was observed that the prevalence of HBV infection was significantly higher in the first half of the study period compared to the second half and higher in the low socioeconomic status group. Conversely, the prevalence was significantly lower in the adolescent group compared to the other age groups. No significant differences in the prevalence of HBV infection were found in relation to being a private case, parity, or residential location. The findings of the univariate analysis were confirmed using multivariate analysis, and no other significant risk factors were identified. Note that in the multivariate analysis, as presented in Table 2, the prevalence in the first half of the study was still significantly higher, with an odds ratio of 1.36 (*p*-value < 0.001). In contrast, the weak association of HBV-positive status with low socioeconomic status found in the univariate analysis was not confirmed by multivariate analysis. Within the adolescent group, the probability of HBV infection decreased, with an odds ratio of approximately 0.607 (*p*-value < 0.001).

## 4. Discussion

New insights gained from this study are as follows: (1) The overall prevalence of HBV infection in pregnant women has reduced significantly from 6.11% in 2003 to 3.15% in 2022. (2) The magnitude of reduction was different among age groups; at the end of the study period, the most reduction in prevalence was observed in the adolescent group, contributing to nearly 0% in many recent years, while the prevalence in elderly women remained the same throughout the past two decades. The trend of decrease in the adolescent group was likely associated with the women being born in the era of routine neonatal HBV vaccination. The age of women with HBV infection tended to be significantly higher, with the current average age of pregnant women with HBV infection being 34 years. This further reinforces the efficacy of the HBV vaccine policy. (3) Other than the timeframe of the study and maternal age, other potential risk factors, such as low socioeconomic status, were not significantly associated with the prevalence of HBV infection. The obtained data can contribute to affirming the goals set by the World Health Organization (WHO), suggesting that these objectives are achievable with the continued implementation of preventive policies. This is particularly evident in the absence of HBV infections among pregnant women of younger age groups, indicating positive outcomes from the current preventive measures.

A notable finding from this study is the unchanged prevalence or increasing trend in HBV infection among individuals aged over 35, similar to the observations in the study by Deng Q et al. [22]. This phenomenon might be explained by the fact that individuals in this age group typically did not receive the HBV vaccine at birth or may have received it initially but experienced a decline in immunity over time, making them more susceptible to infection. According to the current ACOG guidelines for managing HBV infection during pregnancy, it is recommended to conduct a triple panel test, including HBsAg, Anti-HBs, and Anti-HBc, in pregnant women who have not been tested for immunity levels after the age of 18. Additionally, vaccination against HBV is advised for individuals lacking immunity [23].

**General comments:** The results presented here demonstrate that the HBV prevention measures were highly effective among our pregnant population, which is also reflective of the general population. Our results indirectly support that vertical transmission is the main route of HBV infection in our population. This conclusion is based on the fact that in the early part of the first decade, women of all age groups in our population were in the era of no immunoprophylaxis in newborns, and they had a comparable high prevalence, while at the end of the second decade in which most women in the adolescent group, some in the adult group, but no one in the elderly group received immunoprophylaxis, the prevalence was nearly 0%, half-reduced, and unchanged, respectively. Note that the prevalence of HBV infection in the elderly group was relatively stable throughout the two decades. The women in this group were born prior to the year 2003 and were expected not to receive neonatal immunoprophylaxis. Nevertheless, horizontal transmission might also be responsible for such a higher rate in the group of advanced age as they had more time for exposure to HBV infection. As a consequence, the trend of higher rates of HBV among women of higher maternal age in more recent years indicates that there is a significant shift in the age group of maternal HBV infection from a lower maternal age to a higher maternal age. This is because of a dramatically decreased prevalence in younger women, especially in adolescent pregnancies, which dropped to nearly zero in recent years, close to the prevalence in adolescents reported in the USA (*2021 Viral Hepatitis Surveillance Report*) [24]. The prevalence in other age groups was still much higher in our study, though it is continuously decreasing in recent years. The unchanged prevalence among elderly pregnant women might simply be explained by the fact that they must have not been vaccinated in neonatal periods because of being born before the launch of routine vaccination.

**Future trends:** Our results suggest that the overall prevalence of HBV infection among our pregnant women will probably be less than 1.0% in the near future. This suggestion is based on the fact that although the overall prevalence reduced from 6.11% in 2003 to 3.15% in 2022, the magnitude of reduction was very different among the different age groups. The percentage of reduction was very striking in the adolescent group, dropping from 8.6% in 2003 to 0% in 2022 or being nearly eradicated in the most recent years. The difference in magnitude of prevalence reduction among age groups is very meaningful. It is reasonable to assume that adolescent women at the present time, who will be in a highly reproductive age group in the near future and account for the greatest proportion of the obstetric population, are very unlikely to be positive for HBV infection, though horizontal transmission can occur with more advanced age. Probably, we expect to have an exponential reduction rather than a linear reduction. In the early part of the first decade or the era of no HBV vaccination in all age groups, the prevalence in the adolescent group was comparable with that in the adult group, but at the end of the second decade, in which most adolescent women received HBV vaccination in their neonatal period but most adult women did not, the prevalence was very different. In the next five years (2027), all of the pregnant women in the adult group will have been born after 1992, implying that most of them should have received neonatal HBV vaccination. Thus, it is reasonable to expect that the prevalence of infected women in the adult and adolescent groups in the era of routine vaccination will be comparable with a prevalence of less than 1% compared with a prevalence of 6–8% in the early part of the first decade. As a consequence, while the prevalence will be very low among pregnant women, new cases of vertical transmission will also be very rare.

**Clinical and policy implications:** Different from most previous studies that report the prevalence trends in the general population, this study focused on the prevalence changes in pregnant women, which is more reflective of the future prevalence trends because vertical transmission is the main route of transmission in our country. The prevalence of HBV in pregnant women is more significantly influenced by the number of new cases in the future than the prevalence in the general population. The prevalence of HBV in pregnant women can play an important role in predicting the future trend in its own population. As the most effective strategy to prevent new cases is routine neonatal HBV vaccination, early HBIG administration to the newborns of HBV-positive mothers, and antiretroviral drug administration to mothers with high viral levels, the epidemiological data of HBV infection in pregnant women are very crucial to determine preventive measures. Therefore, our results may be used to guide our national or hospital policy in terms of suitable budget distribution or cost-benefit analysis.

**Weaknesses:** The limitations are as follows: (1) Due to the lack of data about the history of partial or full HBV vaccination in the past of each woman, we could not know whether they had HBV infection due to the failure of immunoprophylaxis or not. (2) The data on neonatal status were not available, and vertical or horizontal transmission could not be assessed. (3) Due to its retrospective nature, the reliability of the data might not be perfect. (4) The results could not be generalized in other countries as the prevalence, route of transmission, and policies of disease control vary from country to country.

**Strengths:** The strengths are as follows: (1) In addition to reflecting the trends of the overall prevalence of HBV infection as reported in most previous studies, the prevalence changes in this study can also help us estimate the prevalence of vertical infection in the near future. (2) An adequate sample size and long timeframe were used to address the primary objective of the study.

**Research implications:** Due to the fact that the prevalence of HBV infection is markedly varied among geographical areas, and there are different routes of transmission, which need different strategies of prevention and control, each population should develop its own data concerning the effectiveness of reducing new cases of HBV infection. Even in our country, other parts of Thailand should also develop their own data to determine whether or not the same trend is present. As our analysis did not incorporate the effectiveness of reducing mother-to-child transmission through antiviral treatment for high-risk pregnant women, given this policy has been in effect for only the past 7 years, further longitudinal studies are necessary to comprehensively evaluate its sustained impact and effectiveness over an extended period.

## 5. Conclusions

The prevalence of HBV infection has significantly decreased in the past two decades from 6.11% in 2003 to 3.15% in 2022. The percentage of reduction was very striking in the adolescent group, dropping from 8.6% in 2003 to 0.0% in 2022 or being nearly eradicated in the most recent years. Our results suggest that the overall prevalence of HBV infection among our pregnant women will probably be less than 1.0% in the near future. Our results may be expected to be reproduced in other parts of Thailand because of the same background characteristics and the same national policy.

## Figures and Tables

**Table 1:** Prevalence by years of HBV infection in total pregnant women and subgroups.

Year	Total Pregnancies			Adolescent Pregnancies			Adult Pregnancies			Elderly Pregnancies		
	No.	HBV +ve	Preva-lence	No.	HBV +ve	Preva-lence	No.	HBV +ve	Preva-lence	No.	HBV +ve	Preva-lence
2003	2750	168	6.11	230	19	8.26	2106	135	6.41	414	14	3.38
2004	2622	146	5.57	204	7	3.43	2012	121	6.01	406	18	4.43
2005	2414	156	6.46	221	19	8.60	1826	117	6.41	367	20	5.45
2006	2007	108	5.38	193	11	5.70	1495	78	5.22	319	19	5.96
2007	2205	123	5.58	184	7	3.80	1695	101	5.96	326	15	4.60
2008	2137	147	6.88	202	9	4.46	1600	121	7.56	335	17	5.07
2009	2216	130	5.87	208	4	1.92	1676	110	6.56	332	16	4.82
2010	2084	128	6.14	166	3	1.81	1586	107	6.75	332	18	5.42
2011	1898	94	4.95	149	7	4.70	1453	74	5.09	296	13	4.39
2012	2057	119	5.79	208	3	1.44	1555	93	5.98	294	23	7.82
2013	1823	106	5.81	175	1	0.57	1376	88	6.40	272	17	6.25
2014	1686	96	5.69	119	2	1.68	1290	81	6.28	277	13	4.69
2015	1567	84	5.36	92	3	3.26	1176	67	5.70	299	14	4.68
2016	1429	61	4.27	76	0	0.00	1103	46	4.17	250	15	6.00
2017	1537	58	3.77	50	0	0.00	1154	43	3.73	333	15	4.50
2018	1518	74	4.87	59	1	1.69	1131	47	4.16	328	26	7.93
2019	1494	53	3.55	35	0	0.00	1081	35	3.24	378	18	4.76
2020	1302	48	3.69	33	0	0.00	966	35	3.62	303	13	4.29
2021	1164	38	3.26	24	0	0.00	838	21	2.51	302	17	5.63
2022	1048	33	3.15	25	0	0.00	745	15	2.01	278	18	6.47

**Table 2:** Univariate and multivariate (logistic regression) analysis of potential risk factors for HBV infection.

	Univariate		Multivariate	
Potential Risk Factors	*p*-Value	Odds Ratio (95% CI)	*p*-Value	Odds Ratio (95% CI)
Private cases	0.446	0.949 (0.828–1.087)	0.524	1.053 (0.898–1.235)
First half of study period	<0.001	1.338 (1.215–1.473)	<0.001	1.358 (1.222–1.509)
Nulliparity	0.115	0.929 (0.849–1.018)	0.917	1.006 (0.906–1.116)
Age group	<0.001		<0.001	
Adolescence	0.001	0.676 (0.536–0.852)	<0.001	0.607 (0.465–0.792)
Elderly gravida	0.434	1.049 (0.930–1.184)	0.896	1.009 (0.884–1.151)
Chiang Mai (residency)	0.096	1.089 (0.985–1.204)	0.064	1.108 (0.994–1.234)
Low socioeconomic levels	0.031	1.112 (1.010–1.225)	0.112	1.089 (0.980–1.211)

## Data Availability

The datasets analyzed during the current study are available from the corresponding author upon reasonable request.
